# Risk profile and HIV testing outcomes of women undergoing community-based testing in San Diego 2008–2014

**DOI:** 10.1038/srep42183

**Published:** 2017-02-06

**Authors:** Susannah K. Graves, Susan J. Little, Martin Hoenigl

**Affiliations:** 1 Division of Infectious Diseases, University of California San Diego, San Diego, California, USA; 2 Section of Infectious Diseases and Tropical Medicine, Department of Internal Medicine, Medical University of Graz, Graz, Austria; 3 Division of Pulmonology, Department of Internal Medicine, Medical University of Graz, Graz, Austria

## Abstract

Women comprised 19% of new HIV diagnoses in the United States in 2014, with significant racial and ethnic disparities in infection rates. This cross-sectional analysis of women enrolled in a cohort study compares demographics, risk behaviour, and sexually transmitted infections (STI) in those undergoing HIV testing in San Diego County. Data from the most recent screening visit of women undergoing voluntary HIV screening April 2008 –July 2014 was used. HIV diagnosis, risk behaviour and self-reported STIs were compared among women aged ≤24, 25–49, and ≥50, as well as between HIV-infected and uninfected women and between Hispanic and non-Hispanic women. Among the 2535 women included, Hispanic women were less likely than other women to report unprotected vaginal intercourse (p = 0.026) or stimulant drug use (p = 0.026), and more likely to report one or fewer partners (p < 0.0001), but also more likely to report sex with an HIV-infected individual (p = 0.027). New HIV infection was significantly more prevalent among Hispanic women (1.6% vs. 0.2%; p < 0.001). Hispanic women were more likely than other women to be diagnosed with HIV despite significantly lower rates of risk behaviour. Culturally specific risk reduction interventions for Hispanic women should focus on awareness of partner risk and appropriate testing.

Although the number of new HIV cases in the US has stabilised in recent years, specific groups remain disproportionately affected. While both prevalence and incidence of HIV are higher among men, women accounted for 19% of new HIV diagnoses and 25% of Stage 3 (AIDS) diagnoses nationwide in 2014^1^. While African American women carry the heaviest burden of HIV in the United States^2^,^3^,^4^,^5^,^6^, Hispanic women are also disproportionately affected with a prevalence 1.6 times that of White women in California, though sex-stratified prevalence among Hispanic people is not available at the county level for San Diego^1^,^7^. Whether this finding may be explained by increased risk behaviour remains unclear.

The objective of this study was to compare demographics, risk behaviour, and sexually transmitted infections (STI) in women in San Diego County undergoing community-based HIV testing with detection of acute and early HIV infection (AEH).

## Results

### Overview of participants

Demographics, self-reported behavioural and exposure risk are reported in Table 1. A total of 2538 women underwent Early Test enrolment and screening between April 2008 and July 2014, and three women were excluded from the analysis because they reported already having a diagnosis of HIV prior to testing. Included participants had a median age of 32 years (IQR 25–44), 39.4% self-identified as White, 36.3% as Hispanic/Latina, 11.8% as Black – African American. Overall 91.7% reported they had previously tested for HIV at a median of 9.3 months (IQR 3.3–25.9) before the current testing encounter. One or fewer sex partners in the last 12 months was reported by 44.6% of participants; women older than 50 reported the fewest partners (median 1, IQR 1–2); and women under 25 reported the highest number of partners (median 1, IQR 1–4; p < 0.001). Overall 84.6% of women reported unprotected receptive vaginal intercourse (URVI) and 7.9% of women reported sex with an HIV-infected partner (more frequent among women 25 years or older *versus* those 24 years or younger).

### HIV test results, associated demographics and risk behaviour

Twenty women (0.7%) tested positive for HIV, and 7 (39%) of these had early stage infection with an estimated date of infection within the last 180 days. Of the 11 HIV + patients for whom data were available, the mean CD4 count at presentation was 348 (SD 179) cells/μL and mean viral load was 55,900 (SD 139,000) copies/mL. Demographic and risk behaviour associated with HIV infection are reported in Table 2. Women with HIV infection were significantly more likely to be Hispanic (82.4% vs. 36.0% p = 0.008), to have had sex with a known HIV-infected individual in the last 12 months (33.3% vs. 7.7%, p = 0.002), and to report a sexually transmitted infection in the last 12 months (23.5% vs. 8.3%, p = 0.047) than HIV-uninfected women. There was also a trend toward a higher proportion of HIV-infected women reporting one or fewer partners in the last 12 months (66.7% vs. 44.5%, p = 0.093). Reported stimulant use and injection drug use (IDU) was similar between HIV-infected and uninfected women, as was unprotected vaginal and anal intercourse, sex with injection drug users and sex with men who have sex with men. Very little exchange of sex for money or drugs was reported in our population, though more than half of participants declined to answer this question.

### Risk behaviours of Hispanic versus non-Hispanic women

Because HIV infection was significantly more prevalent among Hispanic women than other women (1.6% vs. 0.2%; p < 0.001), a comparison of risk behaviours in Hispanic versus non-Hispanic women was performed as summarized in Table 3. Hispanic women reported fewer partners in the last 12 months compared to non-Hispanic women, with a median of 1 (IQR 1–2.25) versus 2 (IQR 1–4) partners respectively, and were more likely to report 1 or fewer partners in the last 12 months compared with non-Hispanic women (56.8% vs. 37.8% p < 0.0001). Though a large majority of women in the study reported unprotected vaginal intercourse (84.6%, Table 1), this was significantly less prevalent among Hispanic women than non-Hispanic women (82.4% vs. 86.1%, respectively, p = 0.026). Hispanic women also reported less stimulant drug use (21.4% vs. 25.5%, p = 0.026) and had a trend toward less injection drug use (4.5% vs. 6.3%, p = 0.067). The only behavioural risk factor that was more prevalent among Hispanic women than non-Hispanic women was sex with an HIV-infected individual (9.7% vs. 7.0%, p = 0.027).

In a sub-analysis of Black versus White women, Black women had slightly fewer partners (median 2 for both, IQR 1–3 for Black and IQR 1–4 for White; Mann-Whitney-U test p = 0.01), and were less likely to report sex with an injection drug user (0.03 vs 0.16), or stimulant substance use (0.17 vs 0.30) or injection drug use (0.02 vs 0.09), all with p < 0.001. Additionally, no difference was found in the proportion of Black versus White women reporting unprotected vaginal intercourse (0.86 vs 0.87, p = 0.60), or sex with an HIV-infected partner (0.06 vs 0.09, p = 0.13).

## Discussion

Among women undergoing HIV testing with our program, 0.7% were found to be HIV-infected and previously unaware of their status. Additionally, Hispanic women were disproportionately impacted, with more than double the HIV prevalence observed in non-Hispanic women, despite significantly less risky behaviour. The observed prevalence (0.7%) of HIV-infected and unaware women in the study is nearly 8-fold higher than the overall prevalence of HIV among women in San Diego of 0.09%^7^, suggesting women who presented for testing are a higher risk group than the general San Diego population. Previous venue-based or respondent-driven sampling studies targeting high risk women of colour in the eastern United States report a high prevalence of unaware HIV-infected women, ranging from 1.4% to 9.8%^8^,^9^,^10^,^11^. We observed a comparably high rate of 1.6% new HIV diagnoses among Hispanic women in our study.

Interestingly, although HIV-infected women reported unprotected intercourse at similar rates to those without infection, there was a trend toward a higher proportion of HIV-infected women reporting no more than one sex partner in the prior year. This is in contrast to previous work reporting high rate of concurrency among high-risk women and their partners^12^. The finding of lower risk behaviour among HIV-infected women in our study appears to be driven by the predominance of Hispanic women, who overall had fewer partners, engaged in significantly less unprotected sex than their non-Hispanic counterparts, reported significantly less stimulant drug use, and had a trend toward less reported IDU as well. However, Hispanic women were significantly more likely to report sex with an HIV-infected partner.

Taken together, these data suggest that the increased risk of HIV infection among Hispanic women presenting for HIV testing at Early Test sites in San Diego is primarily a reflection of their increased likelihood of exposure to HIV-infected men in their network of sexual contacts, rather than higher rates of sexual risk behaviour in the women. This finding is similar to reports in the community of men who have sex with men (MSM), where race has been shown to be a strong predictor of risk even though reported behaviours are similar among the groups^13^,^14^,^15^. Additionally, syndemic factors such as poverty, trauma, substance abuse, homelessness and incarceration as well as San Diego’s border location likely contribute to the high prevalence of undiagnosed infection among Hispanic women^16^,^17^,^18^,^19^,^20^,^21^,^22^,^23^,^24^,^25^,^26^. Similarly, the HPTN 064 study, which aimed to estimate the overall new HIV infection rate in women at risk for HIV in the United States, study subjects perceived their risk as due to structural and contextual factors^27^.

This study is subject to a number of limitations. First, high-risk women were not specifically targeted for recruitment, and this combined with the overall low prevalence of HIV among women in San Diego resulted in a small number of newly identified infections from which to draw comparisons. Other authors studying risk behaviours in US women have used targeted sampling in selected high-risk areas to obtain an enriched sample^8^,^9^,^11^,^28^,^29^ or focused their studies on a known high-risk group^30^,^31^,^32^. However, with more than 2500 women enrolled over 6 years, this is one of the largest HIV screening studies in US women published to date. Also, Hispanic and Black women were slightly over-represented in our sample at 36.3% and 11.8% respectively compared with 33.2% and 5.6% of the overall population in San Diego^33^. Additional limitations of our study include its single-centre retrospective design and reliance on self-reported risk behaviour.

In conclusion, our study showed that Hispanic women presenting for free HIV testing at Early Test sites in San Diego are disproportionately affected by HIV infection, and that this increased risk is not linked to higher risk behaviours in this group but rather higher likelihood of having an HIV-infected partner. Culturally-specific HIV risk reduction interventions for Hispanic women are needed which emphasize awareness of partner-risk, knowledge of partner status and prevention methods for serodiscordant couples, including those trying to conceive, such as antiretroviral therapy to suppress viral load and pre-exposure prophylaxis (PrEP). This could provide Hispanic women with the necessary knowledge, skills and services to protect themselves against HIV.

## Methods

### Study design and population

This is a cross-sectional analysis of a cohort study comprised women from a convenience sample of self-selected adults undergoing voluntary HIV screening (single and repeat testers) in San Diego, California, with the Early Test^34^ between April 2008 and July 2014. During this time period, 2535 women were tested and the most recent testing encounter for each is included in the analysis. Data on MSM^35^,^36^ and transgender women and transgender men^37^ testing with the Early Test have been published previously.

### The Early Test: HIV testing, demographic and risk behaviour data collection

Starting in February 2007, the Early Test program (https://theearlytest.ucsd.edu) offered HIV testing, free of charge, to all adults who presented for testing at multiple sites in San Diego County including the San Diego County Health Department; Christie’s Place (a centre for women and families affected by HIV); the University of California, San Diego (UCSD) Antiviral Research Center; several community health centres; the Lesbian, Gay, Bisexual, Transgender Center; and substance abuse treatment centres. Women were also recruited at Planned Parenthood by flyers and provider referral to our testing sites^34^,^38^,^39^. Demographic as well as sexual risk behaviour, STIs, and substance use for the prior 12 months was recorded on a paper case report form by bilingual study personnel performing interviews in English or Spanish. Survey questions were assessed by the testing staff before each HIV testing encounter^38^.

### Statistical analysis

For statistical analysis, SPSS 23 (SPSS Inc., Chicago, IL, USA) was used. Outcome measures included risk behaviour and STIs reported for the previous 12 months at every testing encounter, as well as HIV diagnoses. Outcome measures were compared among women aged ≤24, 25–49, and ≥50, as well as between HIV-infected and uninfected women and between Hispanic and non-Hispanic women using the chi-squared test (for proportions), the Mann Whitney U test (for continuous variables, such as numbers of partners between two groups), and the Kruskal-Wallis test (for continuous variables, such as numbers of partners among three groups). A p-value < 0.05 was considered statistically significant.

### Ethics approval

The University of California, San Diego (UCSD) Human Research Protections Program approved the study protocol and consent, and the methods were carried out in accordance with the UCSD Institutional Review Board’s approved guidelines and regulations. All study participants provided voluntary, written informed consent before any study procedures.

## Additional Information

**How to cite this article:** Graves, S. K. *et al*. Risk profile and HIV testing outcomes of women undergoing community-based testing in San Diego 2008–2014. *Sci. Rep.* **7**, 42183; doi: 10.1038/srep42183 (2017).

**Publisher's note:** Springer Nature remains neutral with regard to jurisdictional claims in published maps and institutional affiliations.

## Figures and Tables

**Table 1:** Participant characteristics.

Demographic or Risk Behaviour	Data available	All Participants n = 2535	Age ≤ 24 n = 562	Age 25–49 n = 1657	Age ≥ 50 n = 316	p
Race/Ethnicity	2425					<0.001*
Non-Hispanic White		955 (39.4%)	179 (33.5%)	634 (40.1%)	142 (46.1%)	
Hispanic/Latino		881 (36.3%)	216 (40.4%)	563 (35.6%)	102 (33.1%)	
Non-Hispanic Black		285 (11.8%)	53 (9.9%)	193 (12.2%)	40 (12.7%)	
Asian		125 (5.2%)	42 (7.9%)	77 (4.9%)	6 (1.9%)	
Other		179 (7.4%)	44 (8.2%)	116 (7.3%)	19 (6.2%)	
Any previous HIV test	2197	2014 (91.7%)	382 (83.4%)	1406 (94.9%)	227 (88.0%)	<0.001*
Months since last HIV test (median, IQR)	1919	9.3 (3.3–25.9)	6.0 (1.9–12.8)	9.9 (3.3–28.3)	14.8 (4.8–61.3)	<0.001*
#of partners, prior 12 months (median, IQR)	2476	2 (1–3)	2 (1–4)	2 (1–3)	1 (1–2)	<0.001*
One or fewer partners in prior 12 months	2476	1105 (44.6%)	185 (33.7%)	715 (44.3%)	205 (65.7%)	<0.001*
Sex with MSM, 12 months	2047	157 (7.7%)	27 (5.9%)	107 (8.0%)	23 (8.9%)	0.231
Sex with injection drug user, 12 months	2472	266 (10.8%)	58 (10.5%)	191 (11.9%)	17 (5.5%)	0.002*
Sex with HIV-infected partner, 12 months	2461	194 (7.9%)	23 (4.2%)	141 (8.8%)	30 (9.8%)	<0.001*
Sex in exchange for money or drugs, 12 months	1348	28 (2.1%)	9 (2.8%)	17 (2.0%)	2 (1.3%)	0.548
Unprotected Receptive Anal Intercourse	2387	223 (9.3%)	50 (9.2%)	157 (9.9%)	16 (6.3%)	0.177
Unprotected Receptive Vaginal Intercourse	2186	1849 (84.6%)	416 (83.0%)	1232 (84.9%)	201 (85.9%)	0.519
Other sexually transmitted infection (STI)						
Any STI, 12 months	2477	207 (8.4%)	63 (11.4%)	129 (8.0%)	15 (4.8%)	0.003*
Bacterial STI, 12 months	2477	108 (4.4%)	37 (6.7%)	63 (3.9%)	8 (2.6%)	0.008*
Any STI ever	2438	416 (17.1%)	51 (9.4%)	293 (18.5%)	72 (23.2%)	<0.001*
Drug Use						
Injection Drug Use, 12 months	2513	154 (6.1%)	34 (6.1%)	112 (6.8%)	8 (2.5%)	0.008*
Use of non-injection stimulants, 12 months#	2513	611 (24.3%)	142 (25.4%)	429 (26.2%)	40 (12.7%)	<0.001*
Early Test HIV Positive	2535	18 (0.7%)	2 (0.4%)	15 (0.9%)	1 (0.3%)	0.368

^*^Statistically significant p < 0.05.

^#^Stimulants: methamphetamine, cocaine, crack, GHB, ecstasy, ketamine; IQR = interquartile range.

**Table 2:** Prevalence of demographic/behavioural risk factors in women with positive and negative HIV test results.

Demographic or Risk Behaviour	HIV+/total 18/2535	Early Test HIV- n = 2517	Early Test HIV + n = 18	p
Race/Ethnicity	18/2425			0.008*
White non-Hispanic		953 (39.6%)	2 (11.8%)	
Hispanic/Latino		867 (36.0%)	14 (82.4%)	
Black non-Hispanic		284 (11.8%)	1 (5.9%)	
Asian		125 (5.2%)	0 (0%)	
Other		179 (7.4%)	0 (0%)	
Age	18/2535			0.368
≤24 years		560 (22.2%)	2 (11.1%)	
25–49 years		1642 (65.2%)	15 (83.3%)	
≥50 years		315 (12.5%)	1 (5.6%)	
Previous HIV test	17/2197	1999 (91.7%)	15 (88.2%)	0.647
Months since last HIV test (median, IQR)	15/1919	9.4 (3.3–26.0)	1.3 (0.48–12.4)	0.018*
Number of partners, prior 12 months (median, IQR)	18/2476	2 (1–3)	1 (1–2)	0.111
One or fewer partners in prior 12 months	18/2476	1093 (44.5%)	12 (66.7%)	0.093#
Sex with MSM, 12 months	15/2047	156 (7.7%)	1 (6.7%)	1
Sex with injection drug user, 12 months	18/2472	263 (10.7%)	3 (16.7%)	0.433
Sex with HIV-infected partner, 12 months	18/2461	188 (7.7%)	6 (33.3%)	0.002*
Sex in exchange for money or drugs, 12 months	11/1348	28 (2.1%)	0 (0%)	1.000
Unprotected Receptive Anal Intercourse	18/2387	221 (9.3%)	2 (11.1%)	0.682
Unprotected Vaginal Intercourse	17/2186	1835 (84.6%)	14 (82.4%)	0.737
Other sexually transmitted infection (STI)				
Any STI, 12 months	17/2477	203 (8.3%)	4 (23.5%)	0.047*
Bacterial STI, 12 months	17/2477	106 (4.3%)	2 (11.8%)	0.168
Any STI ever	17/2438	413 (17.1%)	3 (17.6%)	1.000
Drug Use				
Injection Drug Use, 12 months	18/2513	152 (6.1%)	2 (11.1%)	0.303
Use of stimulants, 12 months¥	18/2513	608 (24.4%)	3 (16.7%)	0.587

^*^Statistically significant p < 0.05.

^#^Trend that approaches statistical significance.

^¥^Stimulants: methamphetamine, cocaine, crack, GHB, ecstasy, ketamine; IQR = interquartile ratio.

**Table 3:** Prevalence of behavioural risk factors in Hispanic versus other women.

Risk Behaviour	n Hispanic/Total 881/2448	Hispanic Women n = 881	Non-Hispanic Women n = 1567	p
Number partners, prior 12 months (median, IQR)	866/2478	1 (1–2.25)	2 (1–4)	<0.0001*
One or fewer partners in prior 12 months	866/2394	492 (56.8%)	578 (37.8%)	<0.0001*
Sex with MSM, 12 months	743/1977	49 (6.6%)	99 (8.0%)	0.253
Sex with injection drug user, 12 months	864/2389	73 (8.4%)	176 (11.5%)	0.018*
Sex with HIV-infected partner, 12 months	858/2377	83 (9.7%)	107 (7.0%)	0.027*
Sex in exchange for money or drugs, 12 months	449/1302	11 (2.4%)	17 (2.4%)	0.688
Unprotected Receptive Anal Intercourse	819/2306	67 (8.2%)	150 (10.1%)	0.137
Unprotected Vaginal Intercourse	743/2112	612 (82.4%)	1179 (86.1%)	0.026*
Drug Use				
Injection Drug Use, 12 months	872/2427	39 (4.5%)	98 (6.3%)	0.067#
Use of stimulants, 12 months¥	872/2427	187 (21.4%)	396 (25.5%)	0.026*

^*^Statistically significant p < 0.05.

^#^Trend that approaches statistical significance.

^¥^Stimulants: methamphetamine, cocaine, crack, GHB, ecstasy, ketamine; IQR = interquartile range.
